# Twelve-Year Cardiovascular and Mortality Risk in Relation to Smoking Habits in Type 2 Diabetic and Non-Diabetic Men: Tehran Lipid and Glucose Study

**DOI:** 10.1371/journal.pone.0149780

**Published:** 2016-03-01

**Authors:** Farzad Hadaegh, Arash Derakhshan, Amirhossein Mozaffary, Mitra Hasheminia, Davood Khalili, Fereidoun Azizi

**Affiliations:** 1 Prevention of Metabolic Disorders Research Center, Research Institute for Endocrine Sciences, Shahid Beheshti University of Medical Sciences, Tehran, Iran; 2 Endocrine Research Center, Research Institute for Endocrine Sciences, Shahid Beheshti University of Medical Sciences, Tehran, Iran; University of Tampere, FINLAND

## Abstract

**Introduction:**

To examine the associations between smoking and cardiovascular disease (CVD) / coronary heart disease (CHD) and all-cause mortality events in men with and without type 2 diabetes (T2D) in a Middle Eastern cohort during a median follow-up of 12 years.

**Methods:**

The study population included 2230 subjects aged ≥ 40 years, free from CVD, comprised of 367 participants with diabetes (21.2% current smokers) and 1863 without (27.3% current smokers). Multivariate Hazard ratios (HR) and 95% confidence intervals (CI) were calculated for smoking (considering different definitions) for those with and without diabetes. Potential confounding factors including age, body mass index, estimated Glomerular Filtration Rate, hypertension, hypercholesterolemia and educational level were entered in the multivariate analysis.

**Results:**

In men with diabetes, the HR (95% CI) of comparing current and non-smokers was 1.25 (0.74–2.12) for incident CHD, 1.52 (0.96–2.40) for CVD and 2.10 (1.27–3.47) for mortality events; the corresponding values for men without diabetes were 1.65 (1.24–2.20), 1.70 (1.30–2.22) and 1.72 (1.14–2.58), respectively (all P values for interactions > 0.46). After pooling past smokers with current smokers, among diabetic individuals there was no significant risk for CVD [1.29 (0.89–1.86)] or mortality events [1.25 (0.81–1.92)]; however, among non-diabetic individuals the HRs of current/past smokers reached significant levels for CVD [1.53 (1.23–1.91)] but not for mortality outcomes (all P values for interactions > 0.51).

**Conclusions:**

The strength of the associations between smoking habits and incident CVD/CHD and mortality events from all causes did not differ significantly among diabetic and non-diabetic participants. Therefore, a comprehensive community-based smoking prevention program is important, given the increasing trend of smoking among the Iranian population regardless of diabetes status.

## Introduction

Each year over 3.8 million people die of type 2 diabetes (T2D) and its complications worldwide [1]. The occurrence of T2D has risen rapidly over the past decades. Specially, the Middle Eastern countries, which have the highest increasing rates of the disease [2]. In a current study, the annual age-standardized incidence rate of T2D in an Iranian population was reported to be 9.94 per 1000 person-years [2].

One of the most important complications of T2D is cardiovascular disease (CVD) compelling heavy financial burdens to societies [3]. In fact, we showed that in an Iranian population presence of T2D exhibited a coronary heart disease (CHD) risk comparable to non-diabetics with a prior CHD [4]. Other major risk factors of CVD both in diabetic and non-diabetic individuals are hypertension, hyperlipidemia, obesity and smoking [5–7]. The role of smoking as an established risk factor for developing CVD among the general population has been confirmed. Moreover, smoking is highly prevalent in Middle Eastern countries especially in men [8]. A meta-analysis on the prevalence of smoking among Iranian population revealed a 20% prevalence of smoking among Iranian male adults which was shown to be 6.02 times more than their female counterparts [8].

It has been reported that cigarette smoking had a large and significant interaction with diabetes, such that an estimated 65% of the cardiovascular disease deaths among diabetics could be attributed to the interaction of diabetes and cigarette smoking among older Caucasian adults; however, there is conflicting data regarding the issue that the presence of diabetes modifies the impact of smoking on CVD outcomes [9–13]. Therefore, it remains uncertain whether smoking confers excess cardiovascular risk in people with T2D above and beyond what is reported in smokers without T2D, and consequently, whether smoking can account for some of the excess cardiovascular risk observed in diabetic patients. Moreover, as emphasized recently in a meta-analysis by Pan et al. [14], despite horrifying statistics on the population burden of T2D and smoking among Middle Eastern population, data on their dynamics regarding CVD outcomes continues to be lacking,.The objective of the present study was to investigate the associations between smoking and cardiovascular outcomes and all-cause mortality events in men with and without T2D of a Middle Eastern cohort, the Tehran Lipid and Glucose Study (TLGS).

## Methods

### Study population

This study was conducted within the framework of TLGS, a longitudinal population-based ongoing study aimed at determining the risk factors and outcomes for non-communicable diseases. The TLGS has two major components: a cross-sectional prevalence study of non-communicable diseases and associated risk factors, implemented between March 1999 and December 2001, and a prospective follow-up study. Data collection is ongoing, designed to continue for at least 20 years, with examinations at 3-year intervals [15]. From a total number of 2951 men attended phase I (1999–2001) and II (2002–2005), aged 40 years and above, those with a history of CVD at baseline (n = 362) and those with missing data (i.e. diabetes and smoking status and all of the covariates) (n = 178) were excluded, leaving 2411 subjects, of whom 2230 subjects were followed up until 20^th^ March 2012 with a median follow up of 12.05 years (response rate 2230/2951-359 ≈86%). The ethical committee of the Research Institute for Endocrine Sciences (RIES) approved this study and written informed consent was obtained from each subject (S1 File).

### Clinical, anthropometric and laboratory measurements

The participants were interviewed by trained physicians to obtain past medical history and to collect information using a pretested questionnaire including familial history of non-communicable disease, smoking habits, reproductive history and assessment of physical activity [16]. For collecting clinical data, weight was measured while the subjects were minimally clothed and without shoes using digital scales and was recorded to the nearest 100 g. Height was measured in a standing position without shoes using a tape meter while the shoulders were in a normal state. Body mass index (BMI) was calculated as weight in kilograms divided by height in meters squared. Waist circumference (WC) was measured at umbilical, without any pressure to body. To measure blood pressure, subjects were first made to rest for 15 minutes, when a qualified physician took the blood pressure twice in a seated position after one initial measurement for determining the peak inflation level using a standard mercury sphygmomanometer. Thereafter the mean of the two measurements was considered to be the participant’s blood pressure. A blood sample was taken after 12 to 14 h of overnight fasting for biochemical measurements. Biochemical analysis was conducted on fasting plasma samples; all blood analyses were carried out at the TLGS research laboratory on the day of blood collection. For the oral glucose tolerance test, 75 g glucose was administered orally to the subjects who did not use anti-diabetic medications and plasma glucose was measured 2 h after. Total cholesterol (TC) was assayed using the enzymatic colorimetric method with cholesterol esterase and cholesterol oxidase. Serum creatinine (Cr) levels were assayed by kinetic colorimetric Jaffe method. Analyses were performed using Pars Azmon kits (Pars AzmonInc, Tehran, Iran) and a Selectra 2 auto-analyzer (Vital Scientific, Spankeren, Netherlands). All samples were analyzed when internal quality control met the acceptable criteria. The intra- and inter-assay coefficients of variation were both <2.2% for plasma glucose and 0.5% and 2% for TC, respectively [17]. The details and reference studies for anthropometric and blood pressure measurements as well as quality control in TLGS have been reported elsewhere [16].

### Outcome measurements

Details of cardiovascular outcomes have been published elsewhere [15, 18]. To summarize, each year, in a continuous manner, every participant of the TLGS was followed up for any medical event initially by telephone call; a trained nurse questioned each participant regarding any medical conditions leading to hospitalization in the past year and then, during a home or hospital visit, complementary data were collected by a trained physician. Data collected were then evaluated by an outcome committee consisting of a principal investigator, an internist, an endocrinologist, a cardiologist, an epidemiologist, the physician who collected the data and other invited experts as needed to assign a specific outcome for every event. CHD includes cases of definite myocardial infarction diagnosed by electrocardiography (ECG) and biomarkers (creatine phosphokinase-MB, lactate dehydrogenase, and troponin), probable myocardial infarction (positive ECG findings plus cardiac symptoms or signs and biomarkers showing negative or equivocal results), unstable angina pectoris (new cardiac symptoms or changing symptom patterns and positive ECG findings with normal biomarkers), angiographic-proven CHD, and CHD death. CVD is specified as a composite measure of any CHD events, stroke, or cerebrovascular death.

### Definition of terms

Regarding different classification for smoking status in many studies[19], in the current study we examined the impact of smoking status by three approaches. Participants were grouped as never, past or current smokers. Current smokers were defined as a person who smokes cigarettes daily or occasionally. In addition two different categorization were also conducted: past + never smokers (non-smokers) vs. current smokers and past + current smokers (smokers) vs. never smokers. Type 2 diabetes was defined if the participant was using anti diabetic drugs, or if fasting plasma glucose (FPG) was ≥ 7mmol/L or if the 2-hour post challenge glucose (2-hPCG) was ≥ 11.1 mmol/L[20]. Glomerular filtration rate (GFR) was estimated using the abbreviated prediction equation, provided by the Modification of Diet in Renal Disease study in which, eGFR (estimated GFR) is expressed as mL/min per 1.73 m2 and serum creatinine (Scr) is expressed as mg/dL[21]. Hypertension was defined as systolic blood pressure (SBP) 140mmHg and over or diastolic blood pressure (DBP) 90mmHg or over or current use of antihypertensive medication. Hypercholesterolemia was defined as TC≥ 6.19 mmol/L or current use of lipid lowering drugs. Education had three levels of primary, diploma and higher than diploma.

### Statistical analysis

Findings on covariate variables are expressed as means (SD) or percentages for continuously and categorically distributed variables, respectively. Baseline characteristics regarding different smoking groups were compared by ANOVA for continuous variables with normal distribution, Kruskal–Wallis test for continuous variables without normal distribution and Pearson Chi-square for categorical variables. Cox proportional hazard survival regression model was used to study the association between smoking categories and the outcomes. Survival time was the time from start of the follow-up period to the date of the first incident CVD/CHD or mortality event (failure). The censoring time of an individual was the time from entry into the study to loss to follow-up or the end of the study, whichever happened first. We estimated multivariate-adjusted hazard ratios (HRs) with 95% confidence intervals (95% CIs) for CVD and CHD events and all cause-mortalities. Potential confounding factors including age, BMI, eGFR, hypertension, hypercholesterolemia and educational level were entered in the multivariate analysis. Each model included the above covariates plus smoking groups, considering non-smoking as the reference. Kaplan–Meier analysis was conducted to plot the time-to-event relations for smoking categories in diabetic and non-diabetic groups for each outcome, considering never smoker as reference. We checked interactions between smoking groups and the prevalent diabetes regarding different outcomes in multivariate analyses in a pooled sample, using likelihood-ratio test. The proportional hazards assumption in the Cox model was assessed with the Schoenfeld residual test and all proportionality assumptions were appropriate. Statistical analyses were performed using SPSS for windows version 15 and STATA version 10; p values less than 0.05 were considered statistically significant.

## Results

A sample of 2230 men ≥ 40 years old without a history of CVD in the baseline with mean (±SD) age of 54.69±10.53 years was included in this study among which 367 patients had T2D. During a median of 12.05-year follow-up (interquartile range, 9.8 to 12.5 years), we documented 380 cases of incident CHD (99 cases attributable to diabetic population), 450 cases of incident CVD (123 cases attributable to diabetic population) and 245 deaths from any cause (90 cases attributable to diabetic population).

The baseline characteristics of participants based on smoking groups among diabetics and non-diabetic population are shown in Table 1. There were significant differences in baseline characteristics between different groups of smoking among non-diabetic population excluding for usage of lipid lowering drugs, TC and hypercholesterolemia. Meanwhile, amongst the diabetic patients the only significant differences between the smoking groups were shown to be for SBP and DBP. Also, there was no significant difference in the incidence of CHD, CVD and total mortality events between the smoking groups for both diabetic and non-diabetic populations.

**Table 1:** Characteristics of participants based on cigarette smoking groups and diabetes status. Tehran Lipid and Glucose study (1999–2012).

	Non Diabetic				Diabetic			
	Never smoked (n = 1010)	Past smoker (n = 344)	Current smoker (n = 509)	P-value	Never smoked (n = 211)	Past smoker (n = 78)	Current smoker (n = 78)	P-value
Age	54.95±10.45	56.05±11.17	50.39±9.11	<0.001	59.19±9.44	59.28±9.91	56.56±10.93	0.110
BMI(kg/m^2^)	26.31±3.57	26.20±3.84	25.30±4.28	<0.001	27.66±3.88	27.92±3.72	27.30±4.08	0.606
WC (cm)	91.49±9.93	91.60±10.72	89.28±11.11	<0.001	96.16±10.51	97.67±10.44	95.72±11.17	0.466
eGFR	68.74±11.07	69.01±10.50	72.23±11.17	<0.001	66.87±11.81	67.77±9.96	68.91±10.80	0.382
SBP(mmHg)	126.39±19.91	126.04±19.30	116.38±17.52	<0.001	135.38±23.23	137.17±22.92	127.46±19.50	0.012
DBP(mmHg)	81.05±11.45	78.95±11.47	75.08±11.14	<0.001	82.87±12.38	82.83±12.80	77.22±11.47	0.002
Antihypertensive drug use	63(6.2%)	25(7.3%)	11(2.2%)	<0.001	25(11.8%)	10(12.8%)	5(6.4%)	0.384
Hypertension	321(31.8%)	106(30.8%)	68(13.4%)	<0.001	95(45%)	37(47.4%)	25(32.1%)	0.091
Lipid-lowering drug use	17(1.7%)	7(2%)	10(2%)	0.881	13(6.2%)	6(7.7%)	7(9%)	0.691
TC (mmol/l)	5.41±1.02	5.41±1.18	5.35±1.14	0.669	5.66±1.22	5.67±1.20	5.63±1.33	0.971
Hypercholesterolemia	220(21.8%)	73(21.2%)	113(22.2%)	0.944	71(33.6%)	24(30.8%)	29(37.2%)	0.697
Education								
Higher than diploma	165(16.3%)	54(15.7%)	72(14.1%)	<0.001	28(13.3%)	10(12.8%)	8(10.3%)	0.754
Diploma	413(40.9%)	135(39.2%)	266(52.3%)		84(39.8%)	26(33.3%)	33(42.3%)	
Primary school	432(42.8%)	155(45.1%)	171(33.6%)		99(46.9%)	42(53.8%)	37(47.4%)	
Incident CVD	163(16.1%)	74(21.5%)	90(17.7%)	0.077	70(33.2%)	26(33.3%)	27(34.6%)	0.973
Incident CHD	140(13.8%)	61(17.7%)	80(15.7%)	0.2	56(26.5%)	23(29.4%)	20(25.6%)	0.843
Total Mortality	82(8.1%)	36(10.5%)	37(7.3%)	0.239	53(25.1%)	15(19.2%)	22(28.2%)	0.408
FPG (mmol/l)*	5.11(4.83–5.49)	5.11(4.77–5.44)	5.05(4.66–5.38)	0.006	7.77(6.49–10.82)	8.08(6.97–9.93)	8.16(6.45–12.74)	0.758
2hPCG(mmol/l)*	5.99(4.94–7.27)	5.99(4.83–7.05)	5.72(4.61–6.66)	<0.001	14.04(11.82–18.59)	15.26(12.21–18.70)	14.49(12.10–17.93)	0.475

Mean± SD are shown for continuous variables and p value is calculated with t-test

*Median (interquartile range) are shown for continuous variables without normal distribution and p value is calculated with Kruskal Wallis test, % is shown for categorical variables with p value according to chi-square; CVD, cardiovascular disease; CHD, coronary heart disease; BMI, body mass index; WC, waist circumference; SBP, systolic blood pressure; DBP, diastolic blood pressure; FPG, fasting plasma glucose; 2hPCG, 2-hour post challenge glucose; TC, total cholesterol; eGFR, estimated glomerular filtration rate.

**Fig 1:**
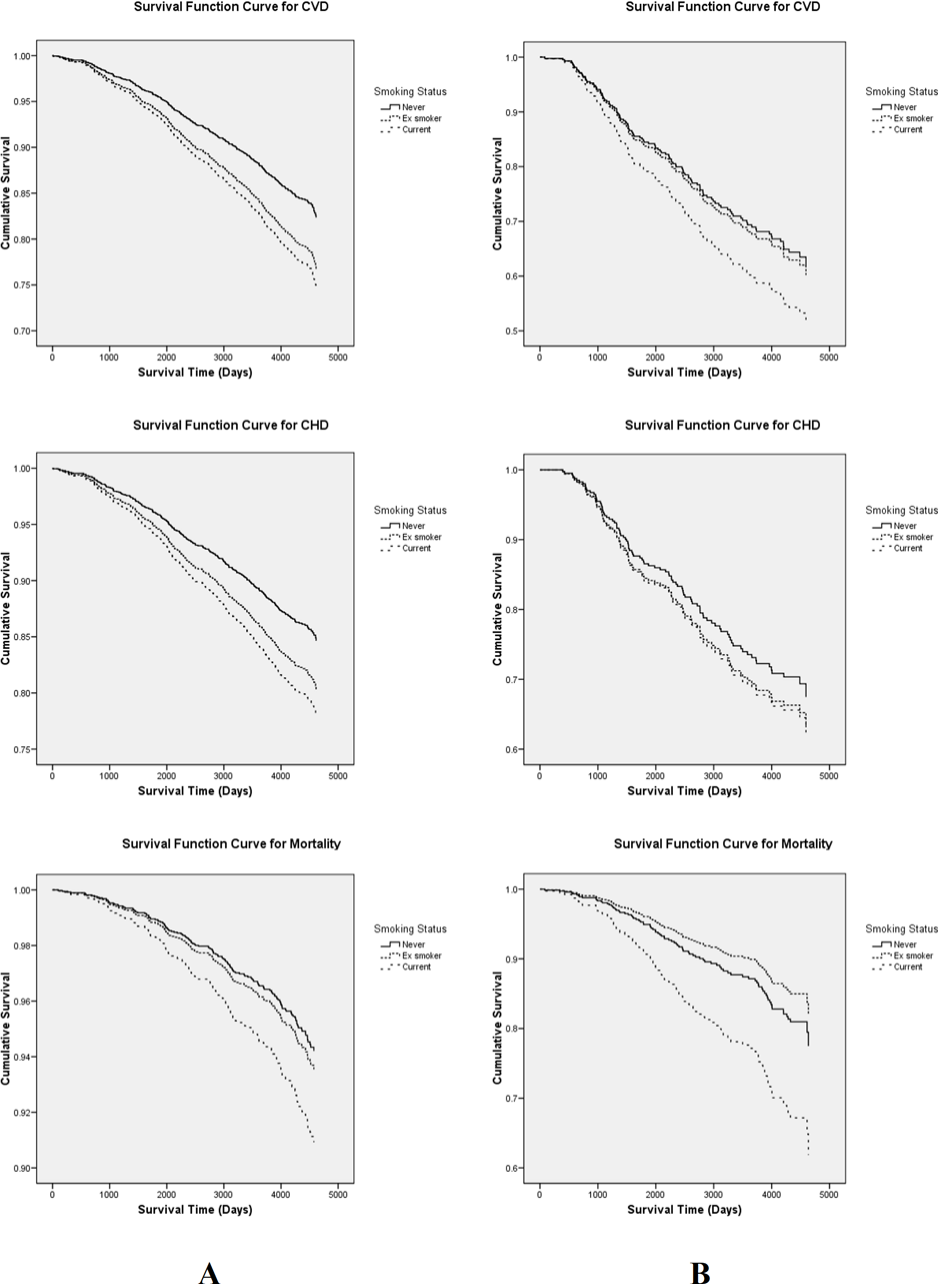
Kaplan–Meier Survival Function Plots of Different Smoking Categories for Incident CVD, CHD and Mortality. (A) Non-diabetic participants, (B) Type 2 Diabetes patients. CVD, cardiovascular disease; CHD, coronary heart disease.

Age and multivariate adjusted hazard ratios (HR) for CVD, CHD and total mortality events for different categories of smoking are presented in Tables 2, 3 and 4. In addition, Kaplan–Meier plots are shown in Fig 1. As illustrated in Table 2, applying 3 categories for smoking definition, among non-diabetics for incident CVD events the HRs of current and past smokers reached significant levels; however, among diabetic population only in the current smoker group we found 52% increased risk which did not reach significant level (HR (95% CI): 1.52 (0.96–2.40), P = 0.076). For CHD events, among non-diabetic population there was significant risk in current smokers while this risk was marginally significant for past smokers (HR (95% CI): 1.34(0.99–1.80)). Regarding mortality events, both diabetic and non-diabetic population showed a significant risk for current smokers. Despite the different hazards in different groups of smoking in diabetic and non-diabetic populations we did not find any significant interactions between smoking and diabetes status for all of the outcomes (all P for interaction > 0.46).

**Table 2:** The risk of different smoking groups between diabetics and non-diabetics participants for cardiovascular and coronary heart diseases and all-cause mortality. Tehran Lipid and Glucose study (1999–2012).

	Diabetic (n = 367)			Non Diabetic (n = 1863)			P value for interaction
	Never smoked	Past smoker	Current smoker	Never smoked	Past smoker	Current smoker	
CVD							
Model 1	1	1.05(0.67–1.65) (p = 0.827)	1.39(0.88–2.18) (p = 0.154)	1	1.36(1.03–1.80) (P = 0.027)	1.50(1.16–1.96) (P = 0.002)	0.667
Model 2	1	1.12(0.71–1.77) (p = 0.634)	1.52(0.96–2.40) (p = 0.076)	1	1.39(1.05–1.83) (P = 0.021)	1.70(1.30–2.22) (P<0.001)	0.753
Mortality							
Model 1	1	0.77(0.43–1.37) (p = 0.375)	1.88(1.14–3.12) (p = 0.014)	1	1.12(0.75–1.66) (P = 0.58)	1.59(1.07–2.37) (P = 0.021)	0.469
Model 2	1	0.81(0.45–1.45) (p = 0.478)	1.99(1.19–3.35) (p = 0.009)	1	1.08(0.72–1.61) (P = 0.712)	1.72(1.14–2.58) (P = 0.009)	0.533
CHD							
Model 1	1	1.17(0.72–1.90) (p = 0.53)	1.20(0.71–2.01) (p = 0.493)	1	1.31(0.97–1.78) (p = 0.075)	1.50(1.13–1.99) (p = 0.005)	0.888
Model 2	1	1.21(0.74–1.98) (p = 0.45)	1.25(0.74–2.12) (p = 0.401)	1	1.34(0.99–1.80) (p = 0.059)	1.65(1.24–2.20) (p = 0.001)	0.898

Model 1: adjusted for age; Model 2: adjusted for age, body mass index, hypertension, hypercholesterolemia, education level and eGFR.

P value for interactions between smoking groups and the prevalent diabetes regarding different outcomes was calculated in multivariate analyses in a pooled sample, using likelihood-ratio test.

**Table 3:** The risk of different smoking groups between diabetics and non-diabetic participants for cardiovascular and coronary heart diseases and all-cause mortality after pooling never and past smokers in a single category. Tehran Lipid and Glucose study (1999–2012).

	Diabetic (n = 367)		Non Diabetic(n = 1863)		P value for interaction
	Never/Past	Current	Never/Past	Current	
CVD					
Model 1	1	1.37(0.89–2.11) (p = 0.155)	1	1.38(1.08–1.78) (P = 0.011)	0.844
Model 2	1	1.47(0.95–2.29) (p = 0.086)	1	1.56(1.21–2.02) (P = 0.001)	0.879
Mortality					
Model 1	1	2.01(1.23–3.28) (p = 0.005)	1	1.55(1.06–2.26) (P = 0.024)	0.444
Model 2	1	2.10(1.27–3.47) (p = 0.004)	1	1.69(1.14–2.50) (P = 0.009)	0.419
CHD					
Model 1	1	1.15(0.70–1.89) (p = 0.589)	1	1.40(1.07–1.82) (p = 0.014)	0.716
Model 2	1	1.19(0.72–1.98) (p = 0.503)	1	1.53(1.17–2.01) (p = 0.002)	0.673

Model 1: adjusted for age; Model 2: adjusted for age, body mass index, hypertension, hypercholesterolemia, education level and eGFR.

P value for interactions between smoking groups and the prevalent diabetes regarding different outcomes was calculated in multivariate analyses in a pooled sample, using likelihood-ratio test.

**Table 4:** The risk of different smoking groups between diabetics and non-diabetics for cardiovascular and coronary heart diseases and all -cause mortality, after pooling current and past smokers in a single category. Tehran Lipid and Glucose study (1999–2012).

	Diabetic (n = 367)		Non Diabetic(n = 1863)		P value for interaction
	Never	Current/Past	Never	Current/Past	
CVD					
Model 1	1	1.20(0.84–1.71) (p = 0.327)	1	1.44(1.15–1.78) (p = 0.001)	0.514
Model 2	1	1.29(0.89–1.86) (p = 0.175)	1	1.53(1.23–1.91) (P<0.001)	0.556
Mortality					
Model 1	1	1.18(0.77–1.80) (p = 0.437)	1	1.32(0.96–1.81) (p = 0.087)	0.713
Model 2	1	1.25(0.81–1.92) (p = 0.314)	1	1.32(0.96–1.82) (p = 0.084)	0.847
CHD					
Model 1	1	1.18(0.79–1.76) (p = 0.413)	1	1.41(1.12–1.79) (p = 0.004)	0.602
Model 2	1	1.23(0.82–1.85) (p = 0.320)	1	1.49(1.18–1.89) (p = 0.001)	0.644

Model 1: adjusted for age; Model 2: adjusted for age, body mass index, hypertension, hypercholesterolemia, education level and eGFR.

P value for interactions between smoking groups and the prevalent diabetes regarding different outcomes was calculated in multivariate analyses in a pooled sample, using likelihood-ratio test.

As shown in Table 3 after merging past smokers with never smokers, among diabetic individuals current smokers were shown to had a twofold increase in risk for total mortality outcome (HR (95% CI): 2.10 (1.02–3.47)); while, among non-diabetics the HRs of current smokers reached significant levels for CVD, CHD and total mortality events with HRs ranged from 53% to 69% (all P < 0.05). On the other hand, after pooling past smokers with current smokers (Table 4), given the subjects who never smoked as reference, among diabetic individuals there was no significant risk for different outcomes; in the meantime, among non-diabetic individuals the HRs of current/past smokers reached significant levels for CVD and CHD events and not for mortality outcomes (Table 4). Finally, when we categorized smoking status in two groups as mentioned in Tables 3 and 4, we also did not find any significant interaction between smoking status and the prevalent diabetes for different outcomes.

## Discussion

In the current study we have examined the combined impact of different diabetes and smoking statuses on incident CVD/CHD and all-cause mortality events during more than 12 years follow-up. Applying 3 categories for definition of smoking status, among diabetic population only the current smoker showed twofold increased risk for mortality events however; among non-diabetics, current smokers showed significant risk for CVD/CHD and mortality outcomes. The absence of any interactions between diabetes status and different smoking categories highlighted that presence or absence of diabetes did not affect the impact of smoking categories on all the outcomes.

The reported prevalence of smoking among Iranian male population (about 20%) [8] was higher than those for Sweden (8.8%) but lower than Chinese (52.9%) and United States (21.6%) males [22–25]. Furthermore, we showed a dramatic increase in trends of cigarette smoking among Iranian population which is inconsistent with previous reports of other populations [7, 26, 27]. Meanwhile, Middle-East has the highest prevalence and incidence of diabetes globally [28]. This is mostly due to nutrition transition, significant increase in prevalence of obesity and the sedentary lifestyle as a consequence of urbanization [29–32].

Relatively few studies in the past have evaluated the interaction between smoking and diabetes on the risk of cardiovascular outcomes [11, 12, 33–37]. These studies have yielded conflicting results which might be attributable to low statistical power of the studies or inadequate level of adjustment. Suarez et al.[9] reported interaction between smoking and diabetes for CVD outcome, this is while, Ford et al. [34] in the National Health and Nutrition Examination Survey showed no interaction between smoking and diabetes for the risk of CVD and mortality.As supported by our data analysis, an individual participant data meta-analysis in men in the Asia Pacific region showed that the effects of cigarette smoking and smoking cessation are broadly similar in men with and without diabetes [38]. Therefore, it might be concluded that the identified risks for smoking among diabetic population are at least equivalent to those found in the general population.

Other studies among diabetic population consistently show that smokers have an increased risk of CVD, premature death, and the microvascular complications of T2D [39]. Despite the 95 percent confidence intervals of the Hazard ratios of current and past smoking included l.0 in the separate models for persons with T2D, the sizes of the coefficients were relatively similar to those for persons without diabetes, suggesting that current and past smoking contribute similarly to the risk of CHD/CVD events among persons with T2D. The reduced power to detect differences among persons with T2D most likely accounts for the lack of statistical significance of current smoking in different definitions. In line with other prospective studies, our data analysis showed that among diabetic population current smoking, excluding when combined with past smoking in a single definition, was associated with more than 2 fold increase risk for all-cause mortality [34, 40].

During a decade follow-up among TLGS population, we recently highlighted that among male population, the rate of current smoking increased from 20 to 24% and from 25 to 35% for diabetic and non-diabetic population, respectively [26]. This is in contrast with previous studies of the other populations [7, 27, 41, 42]. If ignored, increases in the rates of cigarette smoking among Tehranian adult population places both diabetic and non-diabetic populations at intensely increased risk of incident CVD [43]. We previously have shown that among general Iranian population being a past smoker increased the risk of CVD events more than 2 folds [43]. Despite, the absence of any significant interaction between smoking status and presence of diabetes, we highlighted that only among non-diabetic population being past smokers was associated with significant risk for CVD and CHD outcomes this is while; regarding diabetic population we did not see any significant risk among past smokers for different outcomes. Furthermore, combining past with current smokers resulted in non-significant risk of smoking for mortality outcome. However, Qin et al showed a significant relative risk of 48% of mortality events for smokers (included both former and current smokers) [19]; furthermore, they highlighted significant risk for past smokers regarding mortality but not CHD events. These results showed former smokers might still have suffered from a remaining negative impact attributable to smoking but the risk started to decrease [14, 19].

Smoking demonstrates a greater impact on morbidity and mortality than any other physiologic disturbances in patients with T2D. Recently, a meta-analysis among 130,000 diabetic patients demonstrates that smoking was associated with a 36–54% excess risk of mortality for different vascular events including CVD mortality, CHD, stroke and MI [19]. In fact the smoking cessation, with a number needed to treat (NNT) of 11 over 10 years, is associated with the greatest benefit for survival of diabetic patients compared with controlling of other CVD risk factors [44, 45].

Some limitations of the current study needed to be addressed. First, passive smokers were not included in the analysis. The association between passive smoking and risk of CHD events has been well studies [46–48]. Moreover, as the cardiovascular system of a passive smoker lacks the power to provide an appropriate protective response, passive smoking may put the individual at more risk than an active smoker [49]. Second, despite the increasing trend of water pipe smoking prevalence in Iran, it was excluded in the current study. Prevalence of water pipe smoking increased from 35.5% to 40.9% in males aged 10–18 years from 2003–2005 [50]. Third, our study was limited to men over 40 years; therefore the results obtained in the current study might not be applicable to certain age groups especially younger subjects (less than 40 years) and also female populations. Furthermore, our subjects were selected from a sample of Iranian population and further studies should be conducted to determine whether our findings are applicable to other populations and ethnicities. Fourth, we did not have any data regarding the nutritional status of the study population at the baseline of the study, however, the true assessments of these measures can be very difficult to achieve with adequate precision [51]. Lastly, there exist different inter-regional variations of CVD and other cause mortalities due to the smoking epidemics which itself may be affected by different socioeconomic determinants of smoking [52]. On the other hand, the study has some notable strengths. Firstly, to our knowledge this is the first prospective observational study which investigated the impact of smoking for CVD and all-cause mortality events in the presence or absence of diabetes in a Middle-Eastern cohort during more than a decade follow-up. Second, the present study is a population-based cohort with accurate and valid data on risk factors and outcomes.

To conclude, our analysis of the Iranian adult men during more than a decade follow-up shows that the strength of the associations between smoking habits with incident CVD/CHD and mortality events from all causes did not differ significantly among diabetic and non-diabetic participants, although the strength of the association is modest but not significant among diabetic population for CVD events. Therefore, a comprehensive community-based smoking prevention program is important given the increasing trend of smoking among the Iranian population regardless of diabetes status.

## Supporting Information

S1 File STROBE checklist for cohort studies. (DOC)
